# NHCs Catalyzed Hydrophosphonylation of **α**-Ketoesters and **α**-Trifluoromethyl Ketones

**DOI:** 10.1155/2013/890187

**Published:** 2013-12-31

**Authors:** Lin He, Zhi-Hua Cai, Ji-Xin Pian, Guang-Fen Du

**Affiliations:** Key Laboratory for Green Processing of Chemical Engineering of Xinjiang Bingtuan and School of Chemistry and Chemical Engineering, Shihezi University, Xinjiang 832000, China

## Abstract

N-Heterocyclic carbenes catalyzed hydrophosphonylation reaction of **α**-ketoesters and **α**-trifluoromethyl ketones was developed. Under the catalysis of 10 mol% IPr, **α**-ketoesters or **α**-trifluoromethyl ketones reacted with dialkyl phosphites to provide quaternary **α**-hydroxyphosphonates in good to excellent yield.

## 1. Introduction

*α*-Hydroxyphosphonates and phosphonic acids are ubiquious synthons in the synthesis of pharmaceutically and biologically active molecules [1–3]. Hydrophosphonylation of carbonyl compounds catalyzed by base, metal catalysts, or organocatalysts, which is also named as Pudovik reaction, provides facile access to this vital class of compounds [4–7]. However, in contrast to the hydrophosphonylation reaction of aldehydes [8–14], the similar coupling reaction of ketones was scarcely developed [15–22], which may be attributed to the relatively low reactivity of ketones. Therefore, the development of highly efficient catalysts for ketone that participated in Pudovik reaction is still desirable, which will provide *α*-hydroxyphosphonates with a quaternary carbon center.

As an important type of organocatalyst, N-heterocyclic carbenes (NHCs) have been used widely in a series of organic transformations [23–26], such as umpolung and extended umpolung reaction based on ambiphilicity of NHCs [27–30] and transesterification [31–34], formal cycloadditions [35, 36], and other reactions based on nucleophilicity of NHCs. On the other hand, NHCs are organocatalysts that possess strong basicities, and based on this property, only very limited reactions were reported [37, 38]. Recently, we found that NHCs can catalyze the coupling reaction between phosphites and imines (or aldehydes) [39, 40], which inspired us to explore the hydrophosphonylation reaction of ketones with NHCs catalysis.

The study commenced with the reaction of methyl phenylglyoxylate **6a **and dimethyl phosphite **7a** (Table 1). To our delight, under the catalysis of 10 mol% NHC **1 **(1,3-bis(2,6-diisopropylphenyl)imidazol-2-ylidene, IPr) [41], the reaction proceeded very smoothly in CH_2_Cl_2_ to give the desired quaternary *α*-hydroxyphosphonate **8a **quantitatively (Table 1, entry 1). And after the screening of catalysts, base, solvent, and catalyst loading, the optimal reaction conditions were established: using 10 mol% IPr as catalyst, conducted the reaction in dichloromethane at room temperature (Table 1, entry 1).

The reaction scope was then investigated under the optimized reaction conditions and the results were summarized in Table 2. Methyl or ethyl phenylglyoxylate reacted with dimethyl phosphite smoothly to furnish the corresponding *α*-hydroxyphosphonates in high yield. Both electron-withdrawing (-F, -Cl, and -Br) and electron-donating (-OMe) groups that substituted ethyl phenylglyoxylates were all suitable reactants for the coupling reaction, providing the desired products in high yield (Table 2, entries 3–6). Ethyl pyruvate was also good candidate for the addition, affording alkyl-substituted *α*-hydroxyphosphonate **8g** in 90% yield (Table 2, entry 7). Trifluoromethyl ketones, another important type of carbonyl compounds that was used widely in the synthesis of fluorinated molecules, were also tested for the reaction. Experiment results indicated that dimethyl phosphite can add to trifluoromethyl ketones smoothly to give trifluoromethyl-substituted *α*-hydroxyphosphonates in good yields (Table 2, entries 8–10). However, when acetophenone was used instead of *α*-ketoesters or trifluoromethyl ketones, no desired product was obtained and the starting substrates were recovered completely; these results may be attributed to the low reactivity of acetophenone (Table 2, entry 11).

**Scheme 1:**
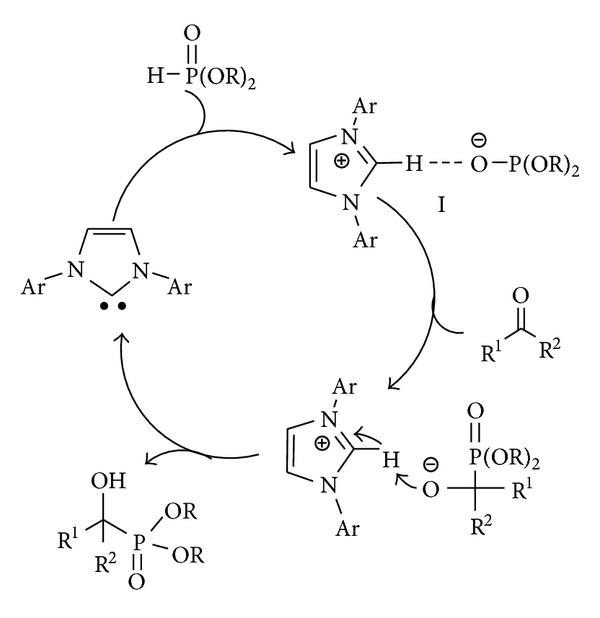
Proposed reaction mechanism.

Based on the previous study of NHCs catalyzed hydrophosphonylation reaction [39, 40], a possible mechanism is proposed in Scheme 1. A complex **I** is formed via the deprotonation of dialkyl phosphite by the basic NHCs catalyst, which might trigger the subsequent coupling of carbonyl compounds and after proton transfer, the desired product will be obtained.

In summary, we have demonstrated an efficient NHCs-promoted hydrophosphonylation of *α*-ketoesters and *α*-trifluoromethyl ketones, which provide a valuable approach for the preparation of quaternary *α*-hydroxyphosphonates.

## 2. Experimental

Unless otherwise indicated, all reactions were conducted under nitrogen atmosphere in oven-dried glassware with magnetic stirring bar. Column chromatograph was performed with silica gel (200~300 mesh) and analytical TLC on silica gel 60-F254. ^1^H NMR (400 MHz) and ^13^C NMR (100 MHz) spectra were recorded on a Bruker-DMX 400 spectrometer in CDCl_3_, with tetramethylsilane as an internal standard and reported in ppm (*δ*). Infrared spectra were recorded on a Nicolet FT/IR-360 spectrophotometer and reported as wave number (cm^−1^). Other starting materials were obtained from commercial supplies and used as received. Anhydrous THF, toluene, Et_2_O, and DCM were distilled from sodium or calcium hydride. Petroleum ether (PE), where used for flash column chromatography, has a boiling range of 60–90°C.

*General Procedure for Preparing of *α*-Hydroxyphosphonates* ***8***. To an oven-dried Schlenk tube were added aldehyde **7** (0.3 mmol), dry dichloromethane (2.0 mL), and phosphite **6** (0.45 mmol), then cooled to 0°C. IPr (10 mol %) was subsequently added under nitrogen and the mixture was stirred at room temperature until completion of the reaction as indicated by TLC. After completion of the reaction, the mixture was extracted by dichloromethane (3 × 20 mL). The combined organic phase was dried by anhydrous sodium sulfate and concentrated under vacuum. The residue was subjected to flash column chromatography (silica-gel and petroleum/ethyl acetate 2 : 1~1 : 1) to obtain *α*-hydroxyphosphonates.

*Methyl 2-(Dimethoxyphosphoryl)-2-hydroxy-2-phenylacetate* ***8a*** *[15].* Colorless oil, yield 99%; ^1^H NMR (400 MHz, CDCl_3_) *δ*: 7.49–7.38 (m, 5H), 5.77 (d, ^2^ *J*_PH_ = 8.2 Hz, 1H), 3.85 (d, ^3^ *J*_PH_ = 11.4 Hz, 3H), 3.74 (s, 3H), 3.60 (d, ^3^ *J*_PH_ = 11.3 Hz, 3H); ^13^C NMR (100 MHz, CDCl_3_) *δ*: 169.2 (d, *J*_CP_ = 6.0 Hz), 134.8 (d, *J*_CP_ = 6.0 Hz), 129.4, 128.8, 127.2, 76.9 (d, ^1^ *J*_CP_ = 4.0 Hz), 54.8 (d, ^2^ *J*_CP_ = 6.0 Hz), 54.4 (d, ^2^ *J*_CP_ = 6.0 Hz), 52.8, 29.7.

*Ethyl 2-(Dimethoxyphosphoryl)-2-hydroxy-2-phenylacetate* ***8b*** *[15].* Colorless oil, yield 89%; ^1^H NMR (400 MHz, CDCl_3_) *δ*: 7.50–7.45 (m, 2H), 7.41–7.36 (m, 3H), 5.75 (d, ^2^ *J*_PH_ = 8.2 Hz, 1H), 4.28–4.16 (m, 2H), 3.85 (d, ^3^ *J*_PH_ = 11.4 Hz, 3H), 3.61 (d, ^3^ *J*_PH_ = 11.4 Hz, 3H), 1.22 (t, *J* = 7.1 Hz, 3H); ^13^C NMR (100 MHz, CDCl_3_) *δ*: 168.7 (d, *J*_CP_ = 5.0 Hz), 134.9 (d, *J*_CP_ = 6.0 Hz), 129.3, 128.8, 127.2, 76.8 (d, ^1^ *J*_CP_ = 11.0 Hz), 61.9, 54.7 (d, ^2^ *J*_CP_ = 6.0 Hz), 54.3 (d, ^2^ *J*_CP_ = 6.0 Hz), 14.0.

*Ethyl 2-(Dimethoxyphosphoryl)-2-(4-fluorophenyl)-2-hydroxyacetate* ***8c***. Colorless oil, yield 84%; ^1^H NMR (400 MHz, CDCl_3_) *δ*: 7.50–7.44 (m, 2H), 7.08 (t, *J* = 8.7 Hz, 2H), 5.73 (d, ^2^ *J*_PH_ = 8.3 Hz, 1H), 4.30–4.16 (m, 2H), 3.86 (d, ^3^ *J*_PH_ = 11.4 Hz, 3H), 3.63 (d, ^3^ *J*_PH_ = 11.3 Hz, 3H), 1.23 (t, *J* = 7.1 Hz, 3H); ^13^C NMR (100 MHz, CDCl_3_) *δ*: 168.6 (d, *J* = 5.0 Hz), 163.2 (d, ^1^ *J*_CF_ = 248.0 Hz), 130.9 (dd, *J* = 6.0, 3.0 Hz), 129.2 (d, *J* = 8.0 Hz), 115.8 (d, *J* = 22.0 Hz), 76.2 (d, ^1^ *J*_CP_ = 4.0 Hz), 62.0, 54.8 (d, ^2^ *J*_CP_ = 6.0 Hz), 54.4 (d, ^2^ *J*_CP_ = 6.0 Hz), 14.0; HRMS(ESI) Calcd for (C_12_H_16_FO_6_P + Na) 329.0566, found: 329.0569.

*Ethyl 2-(4-Chlorophenyl)-2-(dimethoxyphosphoryl)-2-hydroxyacetate* ***8d***. Colorless oil, yield 96%; ^1^H NMR (400 MHz, CDCl_3_) *δ*: 7.39 (q, *J* = 8.6 Hz, 4H), 5.72 (d, ^2^ *J*_PH_ = 8.4 Hz, 1H), 4.27–4.15 (m, 2H), 3.86 (d, ^3^ *J*_PH_ = 11.3 Hz, 3H), 3.64 (d, ^3^ *J*_PH_ = 11.3 Hz, 3H), 1.23 (t, *J* = 7.1 Hz, 3H); ^13^C NMR (100 MHz, CDCl_3_) *δ*: 168.4 (d, *J* = 5.0 Hz), 135.4, 133.5 (d, *J* = 6.0 Hz), 129.0, 128.5, 76.2 (d, ^1^ *J*_CP_ = 5.0 Hz), 62.1, 54.8 (d, ^2^ *J*_CP_ = 6.0 Hz), 54.4 (d, ^2^ *J*_CP_ = 7.0 Hz), 14.0. HRMS(ESI) Calcd for (C_12_H_16_ClO_6_P + Na) 345.0271, found: 345.0282.

*Ethyl 2-(4-Bromophenyl)-2-(dimethoxyphosphoryl)-2-hydroxyacetate* ***8e***. Colorless oil, yield 85%; ^1^H NMR (400 MHz, CDCl_3_) *δ*: 7.53 (d, *J* = 8.6 Hz, 2H), 7.36 (d, *J* = 8.3 Hz, 2H), 5.70 (d, ^2^ *J*_PH_ = 8.4 Hz, 1H), 4.32–4.14 (m, 2H), 3.86 (d, ^3^ *J*_PH_ = 11.3 Hz, 3H), 3.64 (d, ^3^ *J*_PH_ = 11.3 Hz, 3H), 1.23 (t, *J* = 7.1 Hz, 3H); ^13^C NMR (100 MHz, CDCl_3_) *δ*: 168.3 (d, *J* = 5.0 Hz), 134.0 (d, *J* = 6.0 Hz), 132.0, 128.8, 123.6, 76.2 (d, ^1^ *J*_CP_ = 5.0 Hz), 62.1, 54.8 (d, ^2^ *J*_CP_ = 6.0 Hz), 54.5 (d, ^2^ *J*_CP_ = 6.0 Hz), 14.0. HRMS(ESI) Calcd for (C_12_H_16_BrO_6_P + Na) 388.9766, found: 388.9770.

*Ethyl 2-(Diethoxyphosphoryl)-2-hydroxy-2-(4-methoxyphenyl) Acetate* ***8f***. Colorless oil, yield 93%; ^1^H NMR (400 MHz, CDCl_3_) *δ*: 7.33 (d, *J* = 8.7 Hz, 2H), 6.84 (d, *J* = 8.8 Hz, 2H), 5.63 (d, ^2^ *J*_PH_ = 8.4 Hz, 1H), 4.22–4.00 (m, 4H), 3.97–3.82 (m, 2H), 3.74 (s, 3H), 1.28 (td, *J* = 7.1, 1.0 Hz, 3H), 1.19–1.09 (m, 6H); ^13^C NMR (100 MHz, CDCl_3_) *δ*: 168.0 (d, *J*_CP_ = 6.0 Hz), 159.3, 127.7, 126.3, 113.1, 75.4 (d, ^1^ *J*_CP_ = 5.0 Hz), 63.2 (d, ^2^ *J*_CP_ = 6.0 Hz), 62.9 (d, ^2^ *J*_CP_ = 6.0 Hz), 60.7, 54.2, 15.0 (d, *J*_CP_ = 7.0 Hz), 14.9 (d, *J*_CP_ = 7.0 Hz), 13.0. HRMS(ESI) Calcd for (C_15_H_23_O_7_P + Na) 369.1079, found: 369.1075.

*Ethyl 2-(Dimethoxyphosphoryl)-2-hydroxypropanoate* ***8g*** *[42].* Colorless oil, yield 90%; ^1^H NMR (400 MHz, CDCl_3_) *δ*: 8.64 (br s, OH), 4.95–4.87 (m, 1H), 4.27–4.20 (m, 2H), 3.84 (d, ^3^ *J*_PH_ = 11.3 Hz, 3H), 3.79 (d, ^3^ *J*_PH_ = 11.3 Hz, 3H), 1.57 (dd, ^3^ *J*_PH_ = 6.9, 0.7 Hz, 3H), 1.30 (t, *J* = 7.1 Hz, 3H); ^13^C NMR (100 MHz, CDCl_3_) *δ*: 170.5 (d, *J*_CP_ = 5.0 Hz), 72.0 (d, ^1^ *J*_CP_ = 5.0 Hz), 61.7, 54.7 (d, ^2^ *J*_CP_ = 7.0 Hz), 54.5 (d, ^2^ *J*_CP_ = 6.0 Hz), 19.2 (d, ^2^ *J*_CP_ = 6.0 Hz), 14.1.

*Dimethyl 2,2,2-Trifluoro-1-hydroxy-1-phenylethylphosphonate* ***8h*** *[17].* Colorless oil, yield 57%; ^1^H NMR (400 MHz, CDCl_3_) *δ*: 7.53–7.48 (m, 2H), 7.47–7.41 (m, 3H), 5.61 (dd, ^2^ *J*_PH_ = 10.2, 6.4 Hz, 1H), 3.78 (d, ^3^ *J*_PH_ = 11.4 Hz, 3H), 3.57 (d, ^3^ *J*_PH_ = 11.8 Hz, 3H); ^13^C NMR (100 MHz, CDCl_3_) *δ*: 131.2, 130.3, 128.8, 127.9, 76.3 (dd, ^1^ *J*_CP_ = 34.0, 5.0 Hz), 54.7 (d, ^2^ *J*_CP_ = 6.0 Hz), 54.5 (d, ^2^ *J*_CP_ = 6.0 Hz), 29.7.

*Dimethyl 1-(4-Bromophenyl)-2,2,2-trifluoro-1-hydroxyethylphosphonate* ***8i*** *[17].* Colorless oil, yield 64%; ^1^H NMR (400 MHz, CDCl_3_) *δ*: 7.58 (d, *J* = 8.6 Hz, 2H), 7.37 (d, *J* = 8.6 Hz, 2H), 5.69–5.42 (m, 1H), 3.80 (d, ^3^ *J*_PH_ = 11.4 Hz, 3H), 3.61 (d, ^3^ *J*_PH_ = 11.4 Hz, 3H); ^13^C NMR (100 MHz, CDCl_3_) *δ*: 132.0, 130.2, 129.5, 124.7, 75.6 (dd, ^1^ *J*_CP_ = 34.0, 5.0 Hz), 54.7 (d, ^2^ *J*_CP_ = 6.0 Hz), 54.5 (d, ^2^ *J*_CP_ = 6.0 Hz), 29.7.

*Dimethyl 1-(4-Chlorophenyl)-2,2,2-trifluoro-1-hydroxyethylphosphonate* ***8j*** *[17].* Colorless oil, yield 63%; ^1^H NMR (400 MHz, CDCl_3_) *δ*: 7.46–7.40 (m, 4H), 5.59 (dd, ^2^ *J*_PH_ = 10.2, 6.3 Hz, 1H), 3.80 (d, ^3^ *J*_PH_ = 11.4 Hz, 3H), 3.61 (d, ^3^ *J*_PH_ = 11.4 Hz, 3H); ^13^C NMR (100 MHz, CDCl_3_) *δ*: 136.5, 129.8, 129.3, 129.1, 75.7 (dd, ^1^ *J*_CP_ = 34.0, 5.0 Hz), 54.8 (d, ^2^ *J*_CP_ = 6.0 Hz), 54.5 (d, ^2^ *J*_CP_ = 6.0 Hz), 29.7.

## Figures and Tables

**Table 1:** Screening of reaction conditions^a^.

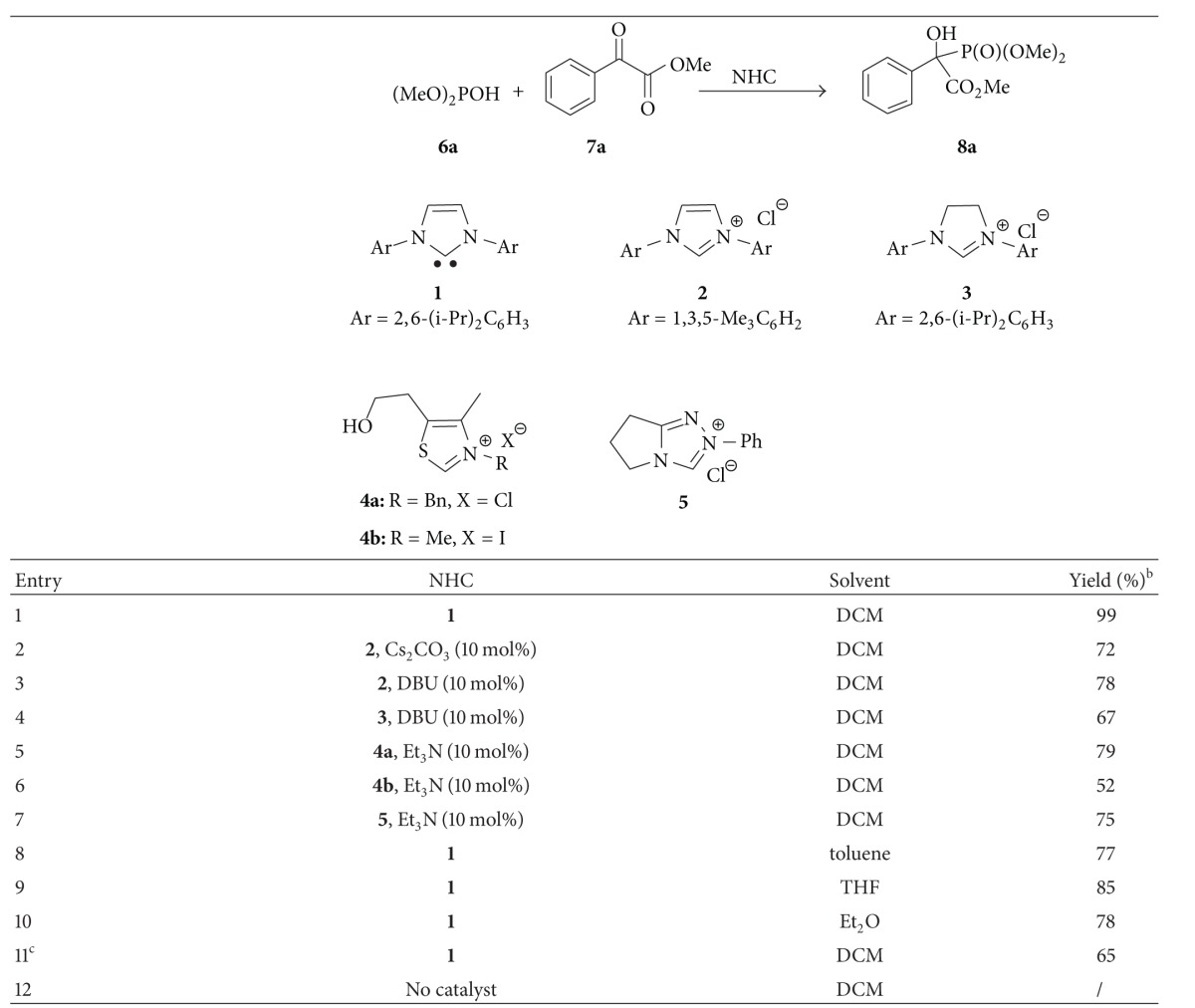

^a^Reaction condition: **6a** (1.5 equiv), **7a** (1.0 equiv), NHC (10 mol%), 0.15 mol L^−1^ of **7a**, and room temperature.

^b^Isolated yield.

^c^Using 5 mol% **NHC 1 **(IPr).

**Table 2:** NHC-catalyzed hydrophosphonylation of phosphites with active ketones^a^.

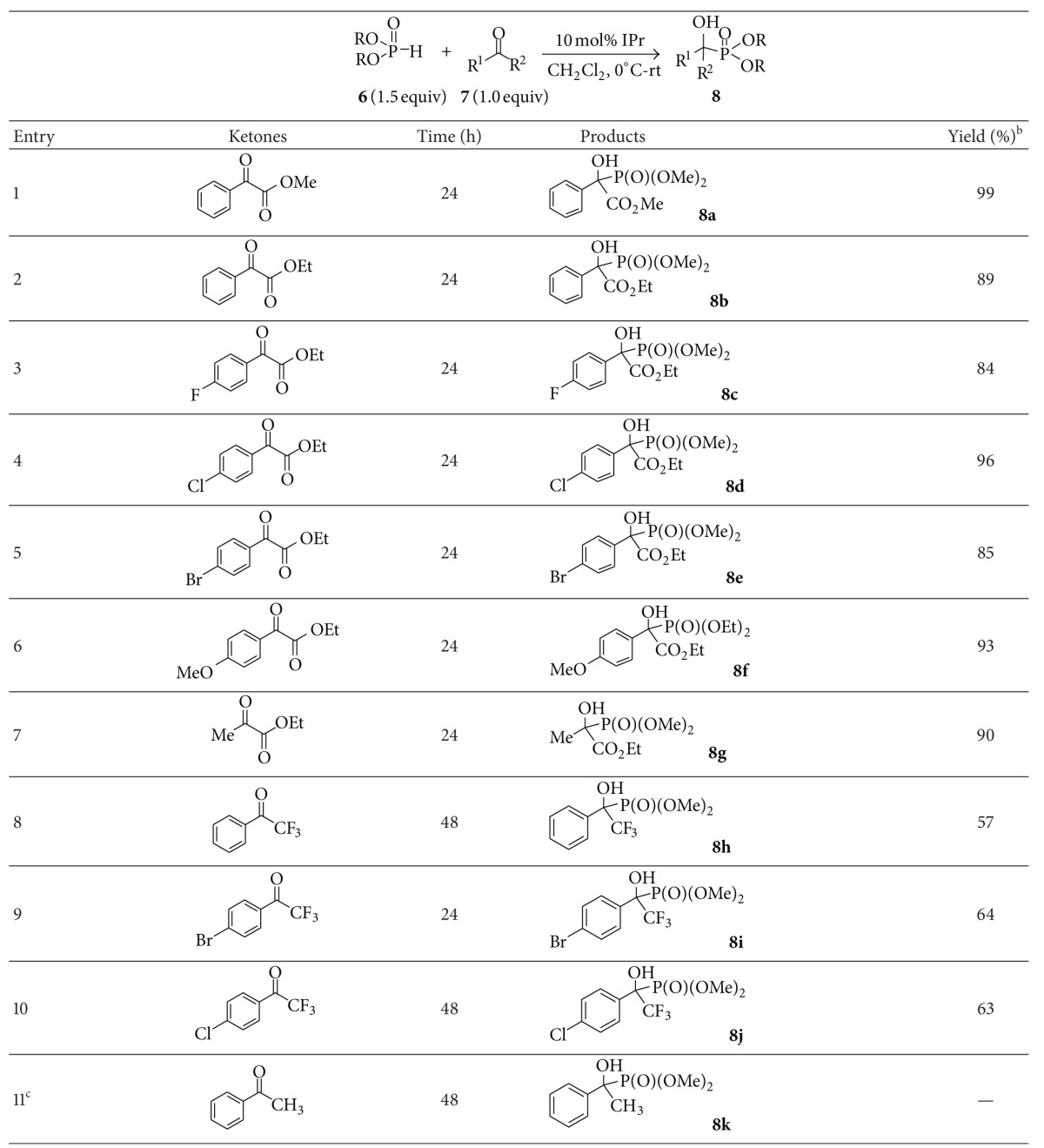

^a^Reaction conditions: **7** (1.5 equiv), **6** (1.0 equiv), IPr (10 mol%), and 0.15 mol L^−1^ of **6**.

^b^Isolated yield.

^c^Recovered yield of acetophenone: 95%.
